# Reply to Lian, M.; Zhang, C. Comment on “Mureșan et al. Prognostic Nutritional Index, Controlling Nutritional Status (CONUT) Score, and Inflammatory Biomarkers as Predictors of Deep Vein Thrombosis, Acute Pulmonary Embolism, and Mortality in COVID-19 Patients. *Diagnostics* 2022, *12*, 2757”

**DOI:** 10.3390/diagnostics15172152

**Published:** 2025-08-26

**Authors:** Adrian Vasile Mureșan, Ioana Hălmaciu, Emil-Marian Arbănași, Réka Kaller, Eliza-Mihaela Arbănași, Ovidiu Aurelian Budișcă, Răzvan Marian Melinte, Vlad Vunvulea, Rareș Cristian Filep, Lucian Mărginean, Bogdan Andrei Suciu, Klara Brînzaniuc, Raluca Niculescu, Eliza Russu

**Affiliations:** 1 Clinic of Vascular Surgery, Mures County Emergency Hospital, 540136 Targu Mures, Romania; 2 Department of Vascular Surgery, George Emil Palade University of Medicine, Pharmacy, Science, and Technology of Targu Mures, 540139 Targu Mures, Romania; 3 Department of Radiology, Mureș County Emergency Hospital, 540136 Targu Mures, Romania; 4 Doctoral School of Medicine and Pharmacy, George Emil Palade University of Medicine, Pharmacy, Sciences and Technology of Targu Mures, 540142 Targu Mures, Romania; 5 Department of Surgery, University of Medicine, Pharmacy, Science and Technology “George Emil Palade” of Targu Mures, 540139 Targu Mures, Romania; 6 Department of Orthopedics, Regina Maria Health Network, 540098 Targu Mures, Romania; 7 Department of Orthopedics, Humanitas MedLife Hospital, 400664 Cluj Napoca, Romania; 8 Department of Anatomy, George Emil Palade University of Medicine, Pharmacy, Science, and Technology of Targu Mures, 540139 Targu Mures, Romania; 9 Department of Pathophysiology, George Emil Palade University of Medicine, Pharmacy, Science, and Technology of Targu Mures, 540139 Targu Mures, Romania

We appreciate the insightful commentary by Mengyi Lian and Chengwei Zhang regarding our manuscript “Prognostic Nutritional Index, Controlling Nutritional Status (CONUT) Score, and Inflammatory Biomarkers as Predictors of Deep Vein Thrombosis, Acute Pulmonary Embolism, and Mortality in COVID-19 Patients” [1,2]. In our research, we found that higher admission levels of inflammatory biomarkers and CONUT Score, along with lower PNI values, were linked to a greater risk of deep vein thrombosis (DVT), acute pulmonary embolism (APE), and mortality among COVID-19 patients [1].

We appreciate Mengyi Lian and Chengwei Zhang for their insightful observations and completely concur with their point that it is unreasonable for all eight variables to become independent risk factors simultaneously [2]. Regrettably, due to a technical oversight, the phrase “multivariate analysis” mistakenly appeared in the final version of the manuscript. Furthermore, the results in Table 5 of the original paper [1], as noted by Mengyi Lian and Chengwei Zhang, actually reflect univariate analysis [2].

In the present response, we include a table illustrating the multivariate analysis in which we propose three adjustment models as follows: Model 1 (age and sex), Model 2 (age, sex, chronic heart failure, atrial fibrillation, myocardial infarction, chronic kidney disease, peripheral arterial disease, malignancy, tobacco, obesity, and dyslipidemia), and Model 3 (age, sex, chronic heart failure, atrial fibrillation, myocardial infarction, chronic kidney disease, peripheral arterial disease, malignancy, tobacco, obesity, dyslipidemia, and all other biomarkers). As demonstrated in Table 1, the association identified in the univariate analysis presented in our manuscript is further corroborated after adjusting for Models 1 and 2. Nevertheless, as articulated by Mengyi Lian and Chengwei Zhan, when accounting for additional biomarkers, the systemic inflammatory index (SII), the aggregate index of systemic inflammation (AISI), and the prognostic nutritional index (PNI) forfeit their predictive capacity concerning DVT. Furthermore, the monocyte to lymphocyte ratio (MLR), platelet to lymphocyte ratio (PLR), SII, systemic inflammation response index (SIRI), and PNI likewise relinquish their predictive significance regarding the risk of APE, while MLR, SIRI, and AISI similarly lose their predictive relevance concerning mortality.

The authors wish to replace the term “multivariate” with “univariate” in the original publication [1]. However, within the current manuscript, we present the multivariate analysis. The authors sincerely apologize for any inconvenience this may cause and state that the scientific conclusions remain unaffected. It is noteworthy to mention that, despite a technical editing error present in our manuscript, which we shall rectify, the results and conclusions of our study continue to hold validity, even after accounting for the presence of comorbidities, as presented in Table 1. In conclusion, we maintain that, despite the editing error we committed, the results and conclusions of our study remain unchanged, are valid, and do not contain any errors that could adversely affect patient management. Consequently, the initial conclusion derived from the study based on univariate analysis is also reaffirmed in the context of multivariate analysis. In conclusion, we sincerely thank Mengyi Lian and Chengwei Zhang for their keen observations and assistance in identifying a technical editing mistake in our original paper.

## Figures and Tables

**Table 1:** Multivariate analysis of predictors for DVT, APE, and mortality during the in-hospital stay.

Biomarkers		Deep Vein Thrombosis			Acute Pulmonary Embolism			Mortality		
		OR	95% CI	*p* Value	OR	95% CI	*p* Value	OR	95% CI	*p* Value
**high-MLR**	Model 1	11.51	7.87–16.82	**<0.001**	6.30	3.55–11.16	**<0.001**	6.80	4.57–10.13	**<0.001**
	Model 2	10.32	6.96–15.29	**<0.001**	4.33	2.33–8.03	**<0.001**	5.73	3.80–8.66	**<0.001**
	Model 3	4.42	7.87–16.82	**<0.001**	1.66	0.70–3.96	0.248	1.83	0.95–3.53	0.070
**high-NLR**	Model 1	11.72	8.01–17.18	**<0.001**	8.98	5.13–15.75	**<0.001**	13.01	8.23–20.51	**<0.001**
	Model 2	10.23	6.88–15.18	**<0.001**	7.70	4.14–14.32	**<0.001**	11.73	7.31–18.85	**<0.001**
	Model 3	2.40	1.31–4.42	**0.005**	3.27	1.32–8.12	**0.011**	2.56	1.19–5.50	**0.016**
**high-PLR**	Model 1	8.53	5.92–12.29	**<0.001**	4.57	2.54–8.24	**<0.001**	10.99	7.29–16.55	**<0.001**
	Model 2	7.85	5.39–11.43	**<0.001**	4.01	2.13–7.55	**<0.001**	10.82	7.02–16.69	**<0.001**
	Model 3	2.24	1.21–4.17	**0.011**	1.06	0.35–3.22	0.917	3.02	1.53–5.96	**0.001**
**high-SII**	Model 1	9.24	6.40–13.87	**<0.001**	5.07	2.78–9.23	**<0.001**	11.34	7.30–17.61	**<0.001**
	Model 2	8.68	5.81–12.97	**<0.001**	4.45	2.32–8.52	**<0.001**	11.26	7.07–17.91	**<0.001**
	Model 3	1.71	0.75–3.91	0.201	0.67	0.17–2.69	0.579	3.07	1.18–7.97	**0.021**
**high-SIRI**	Model 1	9.41	6.42–13.81	**<0.001**	5.66	3.20–10.01	**<0.001**	6.58	4.41–9.81	**<0.001**
	Model 2	8.39	5.62–12.53	**<0.001**	4.39	2.39–8.08	**<0.001**	5.81	3.82–8.81	**<0.001**
	Model 3	2.45	1.09–5.52	**0.030**	1.08	0.31–3.82	0.900	1.61	0.63–4.12	0.320
**high-AISI**	Model 1	6.26	4.39–8.93	**<0.001**	6.02	3.50–10.28	**<0.001**	6.21	4.17–9.25	**<0.001**
	Model 2	5.48	3.81–7.92	**<0.001**	5.28	2.92–9.54	**<0.001**	5.60	3.70–8.47	**<0.001**
	Model 3	0.51	0.23–1.15	0.107	3.36	1.03–11.01	**0.045**	0.70	0.26–1.86	0.476
**high-PNI**	Model 1	5.43	3.81–7.76	**<0.001**	12.75	7.07–22.97	**<0.001**	27.92	16.99–45.89	**<0.001**
	Model 2	5.00	3.47–7.21	**<0.001**	11.58	6.11–21.93	**<0.001**	31.16	18.34–52.95	**<0.001**
	Model 3	1.08	0.63–1.87	0.763	0.61	0.18–2.04	0.417	6.84	3.00–15.59	**<0.001**
**high-CONUT Score**	Model 1	9.72	6.76–13.97	**<0.001**	32.19	16.76–61.82	**<0.001**	41.66	21.95–79.04	**<0.001**
	Model 2	9.16	6.29–13.33	**<0.001**	34.18	16.24–71.86	**<0.001**	44.54	22.91–86.61	**<0.001**
	Model 3	8.24	4.69–14.47	**<0.001**	44.81	12.28–163.39	**<0.001**	10.92	4.24–28.11	**<0.001**

Model 1: age and sex. Model 2: age, sex, chronic heart failure, atrial fibrillation, myocardial infarction, chronic kidney disease, peripheral arterial disease, malignancy, tobacco, obesity, and dyslipidemia. Model 3: age, sex, chronic heart failure, atrial fibrillation, myocardial infarction, chronic kidney disease, peripheral arterial disease, malignancy, tobacco, obesity, dyslipidemia, and other biomarkers.
